# DNA-PKcs participated in hypoxic pulmonary hypertension

**DOI:** 10.1186/s12931-022-02171-x

**Published:** 2022-09-16

**Authors:** Ying-ying Liu, Wei-yun Zhang, Meng-lan Zhang, Yu-ji Wang, Xi-yan Ma, Jung-hong Jiang, Ran Wang, Da-xiong Zeng

**Affiliations:** 1 Department of Pulmonary and Critical Care Medicine, Suzhou Dushu Lake Hospital, Dushu Lake Hospital Affiliated to Soochow University, Medical Center of Soochow University, Suzhou, 215006 People’s Republic of China; 2 Department of Pulmonary and Critical Care Medicine, Changshu No. 2 People’s Hospital, Changshu, People’s Republic of China; 3 Department of Pulmonary and Critical Care Medicine, The First Affiliated Hospital of Soochow University, Medical Center of Soochow University, Suzhou, People’s Republic of China; 4 Department of Respiratory and Critical Care Medicine, The First Affiliated Hospital of Anhui Medical University, Hefei, 230022 People’s Republic of China

**Keywords:** Hypoxic pulmonary hypertension (HPH), Pulmonary vascular remodeling, DNA repair, DNA-PKcs, NOR1, Cell-cycle-related proteins

## Abstract

**Background:**

Hypoxic pulmonary hypertension (HPH) is a common complication of chronic lung disease, which severely affects the survival and prognosis of patients. Several recent reports have shown that DNA damage and repair plays a crucial role in pathogenesis of pulmonary arterial hypertension. DNA-dependent protein kinase catalytic subunit (DNA-PKcs) as a part of DNA-PK is a molecular sensor for DNA damage that enhances DSB repair. This study aimed to demonstrate the expression and potential mechanism of DNA-PKcs on the pathogenesis of HPH.

**Methods:**

Levels of DNA-PKcs and other proteins in explants of human and rats pulmonary artery from lung tissues and pulmonary artery smooth muscle cells (PASMC) were measured by immunohistochemistry and western blot analysis. The mRNA expression levels of DNA-PKcs and NOR1 in PASMCs were quantified with qRT-PCR. Meanwhile, the interaction among proteins were detected by Co-immunoprecipitation (Co-IP) assays. Cell proliferation and apoptosis was assessed by cell counting kit-8 assay(CCK-8), EdU incorporation and flow cytometry. Rat models of HPH were constructed to verify the role of DNA-PKcs in pulmonary vascular remodeling in vivo.

**Results:**

DNA-PKcs protein levels were both significantly up-regulated in explants of pulmonary artery from HPH models and lung tissues of patients with hypoxemia. In human PASMCs, hypoxia up-regulated DNA-PKcs in a time-dependent manner. Downregulation of DNA-PKcs by targeted siRNA or small-molecule inhibitor NU7026 both induced cell proliferation inhibition and cell cycle arrest. DNA-PKcs affected proliferation by regulating NOR1 protein synthesis followed by the expression of cyclin D1. Co-immunoprecipitation of NOR1 with DNA-PKcs was severely increased in hypoxia. Meanwhile, hypoxia promoted G_2_ + S phase, whereas the down-regulation of DNA-PKcs and NOR1 attenuated the effects of hypoxia. In vivo, inhibition of DNA-PKcs reverses hypoxic pulmonary vascular remodeling and prevented HPH.

**Conclusions:**

Our study indicated the potential mechanism of DNA-PKcs in the development of HPH. It might provide insights into new therapeutic targets for pulmonary vascular remodeling and pulmonary hypertension.

## Introduction

Chronic obstructive pulmonary disease (COPD) is a progressive disease characterized by chronic airway inflammation and lung parenchyma destruction [1, 2]. Group 3 pulmonary hypertension (hypoxic pulmonary hypertension, HPH) is the second most common type of PH. It is primarily due to hypoxemia [3, 4]. Recent evidence suggests that 5% to 10% of patients with chronic obstructive lung disease (COPD) are frequently complicated by the development of HPH, with an increased mortality rate [1–4].

Hypoxic pulmonary hypertension (HPH) is a common complication resulting from various chronic respiratory diseases, which is apt to cause poor prognosis of patients [1–3]. Oxygen therapy is the main modality, as there are no effective molecular-targeted drugs for HPH patients in clinical practice [4, 5]. It is urgent to find new therapeutic targets. From a pathological perspective, the main cause of HPH are hypoxia-induced pulmonary vascular constriction and structure remodeling [6–8]. And the pulmonary vascular remodeling under hypoxic condition is the main anatomic factor to induce irreversible pulmonary hypertension [9, 10]. Hence, we need an in-depth study of its pathogenesis, which is of important clinical significance for prevention and treatment of HPH.

DNA damage response (DDR) is a physiological phenomenon in the part of cell metabolism to maintaining genomic stability [11–13]. Besides normal metabolic activities, lots of exogenous factors such as medicines, hypoxia, and inflammation could lead to DNA damage. There are many genes involved in DDR, including checkpoint kinase 1 (CHK1), poly(ADP-ribose) polymerase 1 (PARP1), Pim-1, mitochondrial heat shock protein 90 (HSP90) etc. Several studies have shown specific pharmacological inhibitors like MK-8776 (CHK1 inhibitor), ABT-888 (PARP1 inhibitor), SGI-1776 (PIM1 inhibitor) and gamitrinib (mitochondrial HSP90 inhibitor) down-regulated their target genes, leading to reduced proliferation and increased apoptosis, suggesting their therapeutic potential in PAH [14–18].

DNA double-strand breaks is a deleterious but common form of DNA damage. It can be predominantly repaired by non-homologous end joining (NHEJ) [19]. DNA-PKcs (DNA-dependent protein kinase catalytic subunit, NIH-NCBI gene 5591) as a critical enzyme has been implicated in NHEJ [20, 21]. Recent reports found that the excessive activation of the DNA damage was important for PAH development [16, 22–24]. However, there is insufficient evidence at present to support that DNA repair has a direct causal association with HPH.

In addition, it is well known that Nur77(NR4A1), Nurr1(NR4A2), and NOR1(NR4A3) are members of the NR4A subfamily of orphan nuclear receptors [25, 26]. And previous study showed that NR4A nuclear orphan receptors were associated with DSB repair by promoting DNA-PK assembly at DNA lesions [27]. Meanwhile, other studies have shown that NOR1 may play a crucial role in regulating smooth muscle cells proliferation [28–30]. Our previous and recent studies have proved that NOR1 can promote pulmonary artery smooth muscle cells( PASMCs) proliferation by modulating the expression of cyclin D1 in PAH [31–33]. Some other studies also showed that the cyclin D1 pathway is involved in the regulation of DNA repair in kinds of cells such as cancer cells and vascular smooth muscle cells [34, 35]. But, it is still unknown whether NOR1/ cyclinD1 is related with the progress of DNA repair in PASMCs under the hypoxia condition.

Therefore, we hypothesized that DNA-PKcs affects human PASMC proliferation by regulating NOR1 protein synthesis followed by cyclin D1 and eventually lead to hypoxic pulmonary vascular remodeling in HPH.

## Materials and methods

### Human subject sample collection

This study was approved by the research ethics committee of the First Affiliated Hospital of Soochow University, and written informed consents were obtained from all subjects. In this study, we collected lung tissues from 10 cases of COPD with hypoxaemia and 12 controls patients in our hospital. Human lung samples were obtained from patients who underwent pneumonectomy for lung volume reduction (COPD with hypoxaemia) or lung carcinoma (controls). The explants of pulmonary artery were separated from lung samples of COPD group or control group as described in our previous study [26]. Lung tissue samples and explants of pulmonary arteries were immediately stored in liquid nitrogen. Meanwhile, all participants have been offered with the written informed consent in this recruitment.

### Cell culture

Human PASMCs were kindly donated by the Institute of Respiratory Disease at the Huazhong University of Science and Technology, Wuhan, China. All cells were cultured in DMEM, supplemented with 10% fetal bovine serum (FBS), 1% L-glutamine and antibiotics (Invitrogen, Carlsbad, CA, USA) in 37℃ humidified with the condition of normoxia (21% O_2_, 5% CO_2_) or hypoxia (5% O_2_, 5% CO_2_).

### Transfection

Interfering RNAs targeting DNA-PK and negative control scrambled siRNAs were designed and synthetized by GenePharma (Suzhou, China). Specific interference RNA (siRNA) sequences for DNA-PKcs were selected based on a previous publication [35]. Sequences of siRNAs are as follows: siRNA-DNA-PK sense 5′-GAUCGCACCUUACUCUGUUTTdTdT-3′; siRNA-NC sense 5′UUCUCCGAACGUGUCACGUdTdT-3′.

PASMCs were seeded into 6 well-plates at density of 2 × 10^5^ cells/well.Then cells were transfected with Lipofectamine 2000 (Invitrogen, Carlsbad, CA, USA) following the manufacturers' instruction.

### Cell proliferation analysis

Cell proliferation was measured by using the Cell Counting Kit-8 assay kit (CCK-8, Boster, Wuhan, China) and 5-Ethynyl-2ʹ-deoxyuridine (EdU) assays (RiboBio, Guangzhou, China). According to the manufacturers' instruction, we seeded cells into 96 well-plates with proper density. Cell viability was assessed at 24 h, 48 h and 72 h separately by CCK-8. We aslo used Edu assays kit to evaluate cell viability. Each experiment was performed in triplicate.

### Cell cycle analysis

The effects of DNA damage repair on the cell cycle of PASMCs were examined by Cell Cycle Analysis Kit (Biyuntian, Shanghai, China). PASMCs at a proper density were cultured in 6-well plates in normoxia or hypoxia and with or without transfection for 48 h. Then cells were harvested and fixed in 70% methanol overnight at 4 °C.Then cells were washed with cold PBS twice and stained in propidium iodide (PI)/RNaseA mixture. After incubation in the dark for 30 min at room temperature, cells were analysed by flow cytometry.

### Western blotting

Cells were lysed in ice with RIPA buffer (CST, USA) containing a protease and phosphatase inhibitor for 10 min, and then the protein concentration was measured using the BCA Protein Assay kit (Biyuntian, Shanghai, China). According to the molecular weight of protein, we used different percentages of SDS-PAGE to separate protein samples and transferred them to nitrocellulose membranes. Each membranes were incubated with respective primary antibodies at 4 °C overnight with DNA-PKcs, *p*-DNA-PKcs, cyclin D1 and NOR1 diluted 1:500 (Abcam, USA) and anti-β-actin diluted 1:1000 (Santa Cruz Biotechnology, Santa Cruz, CA, USA). Then, the membranes were washed with Tris-buffered saline with Tween-20 (TBST) for four times, and then incubated with specific HRP-conjugated secondary antibodies for 2 h at room temperature. The protein bands were visualized using an ECL kit.

### Co-immunoprecipitation (Co-IP) assay

Cells were cultured in a 100 mm plate to 95–100% confluence. Then, the cells in each dish were washed twice with cold phosphate-buffered saline (PBS), collected by scraping, and lysed with 1 ml of modified RIPA buffer (Cell Signaling Technology, Danvers, MA, USA) containing protease and phosphatase inhibitor cocktail (Sigma-Aldrich, St. Louis, MO, USA) for 30 min. Cell lysates were collected by centrifugation at 10,000×*g* at 4 °C for 30 min. Clear lysates were pre-cleared by the addition of 50 μl of protein G bead slurry and incubated at 4 °C overnight with rotation. Supernatants were transferred to a new Eppendorf tube and incubated with 1 μg of rabbit anti-DNA-PKcs antibody (Abcam, USA) with rotation overnight in a cold room; this step was followed by an additional incubation for 3–4 h with protein G beads. The beads were washed three times with RIPA buffer and then boiled in 2 × SDS protein loading buffer for 5 min. Samples (20 μl) were loaded on SDS-PAGE gels for western blot analysis.

### qRT-PCR

RNAiso Plus (Takara, Osaka, Japan) was used for extracting total RNA from cell lines and tumor tissues. Reverse transcriptase M-MLV (Takara) was selected for the synthesis of cDNA. Expression values of DNA-PKcs and NOR1 were respectively normalized to the internal controls β-actin. The primer sequences for qRT-PCR of DNA-PKcs, NOR1, β-actin were as follows:

DNA-PKcs: sense 5′-AAAGACTCAAAGCCCCCTGG-3′,

anti-sense5′-GACTGTCACCCGCTCATCAA-3′;

NOR1: sense 5′-CAAGCCTTAGCCTGCCTGTCAG-3′,

anti-sense 5′-GATCTTCCTCAGTTCCACCAGTGC-3′;

β-actin: sense 5′-CACAGAGCCTCGCCTTTGC-3′,

anti-sense 5′-ACCCATGCCCACCATCACG-3′.

Quantitative RT-PCR reactions were performed using SYBR Premix ExTaq™ (Takara) on an ABI Step One Plus Real-Time PCR system (Applied Biosystems, Foster City, CA, USA), according to the operator’s manual.

### Hematoxylin and eosin staining and immunohistochemistry

In hematoxylin and eosin (H&E) assays, tissue samples were immersed in 4% paraformaldehyde for 4 h and transfered to 70% ethanol. Before dehydrated through a graded series of ethanol, biopsy material were placed in processing cassettes.Then biopsy material were embedded in paraffin wax blocks and dewaxed in xylene, rehydrated in ethanol and washed in PBS, followed by staining with hematoxylin and eosin. After staining, sections were dehydrated through increasing concentrations of ethanol and xylene. For immunohistochemistry, tissue samples were fixed in formalin and embedded in paraffin. We used an immunohistochemical detection kit to measure the expression of DNA-PKcs, α-actin and PCNA (proliferating cell nuclear antigen) protein. HPASMCs were seeded in 8-well culture plates plated with cell-climbing slices. Standard immunofluorescence of the cell climbing pieces was carried out. Then, H&E and immunohistochemical staining were observed in ten microscopic fields at × 400 magnification using a fluorescence microscope (Olympus Corporation).

### Pulmonary vascular morphometry

After HE staining and immunohistochemical staining of lung tissue, pulmonary vascular morphometry were performed for pulmonary vascular remodeling. As described previously [35], the vessel wall thickness was expressed as a percentage of the external diameter [(external diameter − internal diameter)/external diameter × 100%]. The assessment was limited to medium and small arteries (≤ 500 μm diameter) with complete circumferential smooth muscle layer. At least ten vessels were measured in each section.

The muscularized vessels ratio was performed basing on the α-actin immunohistochemical staining. All these arteries were classified into three groups: nonmuscularized (25% circumference with α-actin staining), partially muscularized (25 to 75% circumference with α-actin staining) and fully muscularized (75% circumference with α-actin staining).The fully muscularized vessels were calculated and expressed as the percentage of total medium and small arteries.

### Animal models

The rats used in our study were male Sprague–Dawley rats and aged 3–4 months. The rat models were housed in normoxic (21% O_2_) or hypoxic (10 ± 0.5% O_2_) conditions for 28 days in chambers with 21% normoxic conditions (room air containing 21% O_2_ and 78% N_2_) or 10% hypoxic conditions (air containing 10% O_2_ and 89% N_2_) respectively. The rats were randomly divided into normal control group, hypoxic model group, NU7026 treatment group (10 or 50 mg/kg/day), each group consisted of 6 rats. At 24 h post-hypoxia, the experiments rats were daily treated by NU7026 (10 or 50 mg/kg) for 28 days. For regression experiments, rats were housed in hypoxic conditions for 28 days in chambers and then treated with NU7026 (0 or 50 mg/kg/day) for 2 weeks. All injections were administered intraperitoneally (i.p).

### Statistical analysis

All results were expressed as mean ± standard deviation (SD). For the inter-group comparisons, an unpaired two-tailed Student’s t test was applied. For the multi-group comparisons, a one-way ANOVA followed by Tukey’s test as the post hoc test was applied. *P* < 0.05 was considered as significant. Statistical analysis was undertaken by GraphPad Prism 5.0 (GraphPad, San Diego, CA, USA) and SPSS 17.0 software (SPSS, Chicago, IL, USA).

## Results

### Expression of DNA-PKcs is upregulated in HPH tissues

**Fig. 1:**
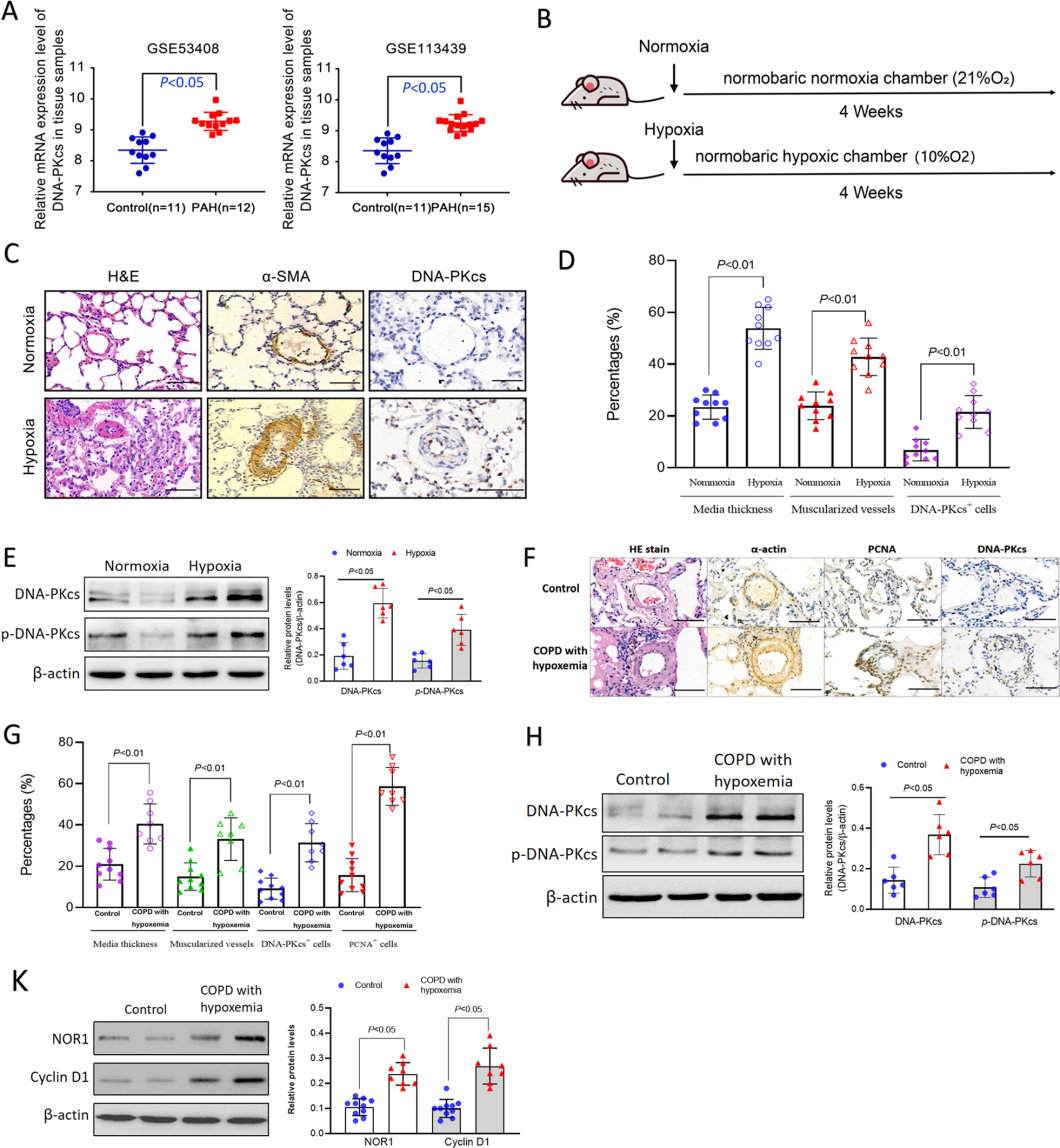
DNA-PKcs up-regulated in lung tissues from HPH rats and COPD patients with hypoxemia. **A** Expression of DNA-PKcs in PAH from GEO database. **B** Establishment of rats HPH models. **C**, **D** Pulmonary vascular remodeling and DNA-PKcs elevation in rat HPH models. **E** Western-blot detection for DNA-PKcs protein levels in explants of rat small pulmonary arteries. **F**, **G** Pulmonary vascular remodeling in lungs from COPD patients with hypoxemia. **H**–**K** DNA-PKcs, NOR1 and cyclin D1 levels in COPD patients with hypoxaemia. Bar 200 µm

As shown in Fig. 1A, the expression of DNA-PKcs from Gene Expression Omnibus (GEO) database was significantly up-regulated in PAH group compared with normal group. Next we used rats models of HPH to detect the hypoxia-induced vascular remodeling and DNA-PKcs expression (Fig. 1B). As expected, H&E staining showed that pulmonary vascular media thickness and muscularized vessels ratio in hypoxic models were significantly higher than controls (Fig. 1C, D). Meanwhile, immunohistochemistry results showed high expressions of the smooth muscle marker smooth muscle α-actin (α-SMA) in lungs tissue sections from hypoxic rats. It also revealed that hypoxia markedly increased the expression of DNA-PKcs in pulmonary vessels in vivo (Fig. 1C, D). Additionally, pulmonary vessel explants were homogenized and we also proved that hypoxia group exhibited high levels of total DNA-PKcs and phosphorylated DNA-PKcs by western blot (Fig. 1E).

All subjects’ baseline characteristics are shown in Table 1. Patients in COPD with hypoxaemia group were older than controls. More patients was smoker or past smokers in COPD with hypoxaemia group. No differences existed between the two groups with regard to age. The forced expiratory volume in 1 s/fixed vital capacity ratio (FEV1/FVC) and forced expiratory volume in 1 s (FEV1% predicted) in COPD with hypoxaemia patients was significantly lower than control group (both *P* < 0.01). The partial pressure of arterial oxygen of COPD with hypoxaemia patients were markedly lower than that in non-COPD patients (*P* < 0.01). We also detected the right heart function using echocardiography. As shown in Table 1, the PASP of COPD patients were significantly higher than that in non-COPD patients (*P* = 0.044), but there was no difference between TAPSE in the two groups (*P* = 0.144).

**Table 1:** The baseline characteristics of patients

	COPD with hypoxaemia	Control	*p* value
N	10	12	
Age	65.90	54.45	0.013
Sex (F/M)	1/9	3/9	0.363
Smoking	8/2	2/10	0.003
FEV1 (%pred)	62.93 ± 10.43	100.47 ± 18.25	< 0.001
FEV1/FVC (%)	57.05 ± 13.45	82.06 ± 17.28	< 0.001
PaO_2_ (mmHg)	65.15 ± 9.43	89.56 ± 12.68	< 0.001
PASP (mmHg)	28.10 ± 4.71	23.42 ± 3.31	0.044
TAPSE	19.20 ± 1.99	20.58 ± 1.93	0.114

*FEV1* forced expiratory volume in first second, *FVC* forced vital capacity, *PASP* pulmonary artery systolic pressure, *TAPSE* tricuspid annular plane systolic excursion

H&E staining demonstrated that intima thickening and luminal stenosis were markedly in COPD with hypoxaemia patients compared with non-COPD patients (Fig. 1F, G). Meanwhile, the expression of DNA-PKcs was higher and proliferation marker PCNA also displayed heightened expression in the COPD with hypoxaemia patients group (Fig. 1F, G). The total DNA-PKcs and phosphorylated DNA-PKcs protein levels in pulmonary arterial explants from COPD with hypoxaemia patients lung tissues were also significantly higher than that in non-COPD patients (Fig. 1H). The NOR1 and cyclin D1 levels were changed similarly in COPD with hypoxaemia patients (Fig. 1K).

### DNA-PKcs promoted hypoxia-induced cell proliferation and cell cycle progression

**Fig. 2:**
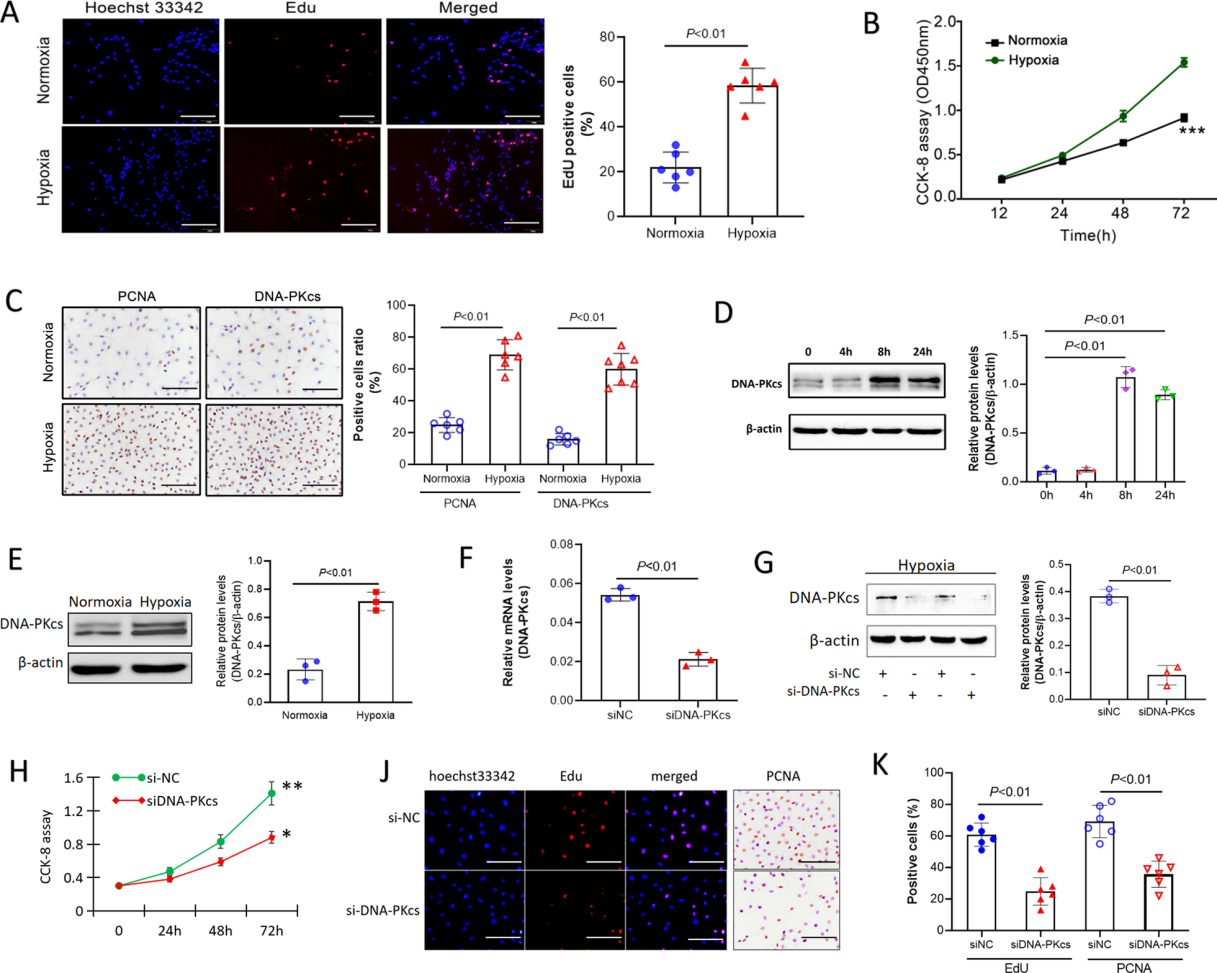
DNA-PKcs specific siRNA suppressed hypoxia-induced PASMCs proliferation. **A** Edu assays and CCK-8 assays for hypoxia-induced proliferation. **B** CCK-8 assay for hypoxic proliferation. **C**, **D** Immunohistochemistry staining and western-blot for expression of PCNA and DNA-PKcs after hypoxia exposure. **E** Elevated DNA-PKcs levels in endothelin cells after hypoxia exposure. **F**, **G** specific siRNA inhibited elevated levels of DNA-PKcs mRNA and protein in hypoxic PASMCs. **H**–**K** Specific siRNA suppressed hypoxia-induced proliferation (CCK-8 assay, Edu assay and immunohistochemistry staining). *P < 0.05, ***P < 0.05, as compared with nomoxia or si-NC. PCNA: proliferating cell nuclear antigen, NC: negative control. Bar 100 µm

To further clarify effects of DNA-PKcs on hypoxia-induced pulmonary vascular remodeling, we evaluated the effect of hypoxia on human PASCMCs growth by CCK-8 assays and Edu assays. Results of Edu assays showed that hypoxia significantly promoted DNA systhesis (Fig. 2A). CCK-8 assays also indicated that hypoxia induced cells proliferation at a time-dependent manner (Fig. 2B). As shown in Fig. 2C, expression of both PCNA and DNA-PKcs was increased in PASMCs exposed to hypoxia. Furthermore, hypoxia promoted DNA-PKcs protein levels at a time-dependent manner (Fig. 2D). Meanwhile, we also evaluated DNA-PKcs expression in endothelial cells. As shown in Fig. 2E, hypoxia significantly up-regulated DNA-PKcs protein levels in endothelial cells as compared with normoxia.

We inhibited DNA-PKcs using specific targeted siRNA in vitro (Fig. 2F). As shown in Fig. 2F and G, specific siRNA markedly prevented the mRNA and protein levels induced by hypoxia exposure in vitro. Moreover, DNA-PKcs specific siRNA not only significantly inhibited hypoxia-induced cells proliferation (CCK-8 assays, shown in Fig. 2H), but also prevented hypoxia-induced DNA systhesis (Edu assays and PCNA immunocytochemistry, shown in Fig. 2J, K).

**Fig. 3:**
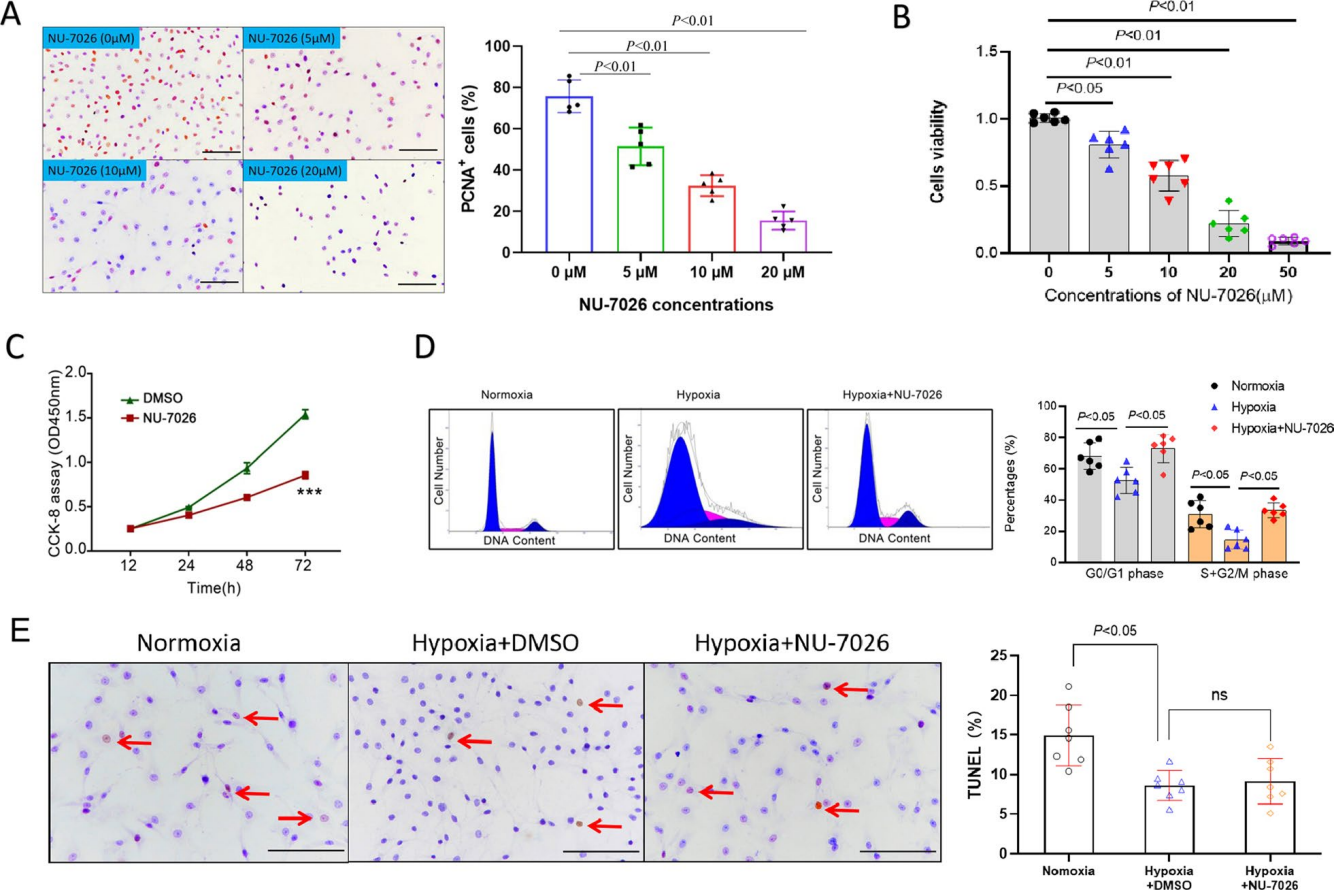
DNA-PKcs specific inhibitor (NU7026) suppressed proliferation and arrested cells cycle in hypoxic PASMCs. **A** Immunohistochemistry staining for PCNA in hypoxic PASMCs after NU7026 treatment with different concentrations. **B**, **C** CCK-8 assays in hypoxic PASMCs after NU7026 treatment with different concentrations or different times. **D** Flow cytometry for cells cycle analysis in hypoxic PASMCs after NU7026 treatment. **E** TUNEL assay in hypoxic PASMCs after NU7026 treatment. Bar 100 µm. (***P < 0.05 as compared with DMSO, ^#^*P* < 0.05)

Next, we blocked DNA-PKcs by the specific small molecular inhibitor NU7026. As shown in Fig. 3A, NU7026 decreased PCNA positive cells under hypoxia condition at a concentration-dependent manner. Cell proliferation also showed reduction by DNA-PKcs specific inhibitor at a concentration-dependent manner (Fig. 3B) and a time-dependent manners (Fig. 3C, CCK-8 assays). Considering experimental efficiency, we selected 10 μM as target concentrations for subsequent experiments.

It is well known that the process of DNA repair is inherently linked to cell cycle progression and its checkpoints. To investigate the potential mechanism of hypoxia in promoting cell growth, we utilized flow cytometry to analyse the distribution of cell cycle. As shown in Fig. 3D, hypoxia significantly decreased proportion of G0/G1 phase cells and raise proportion of S + G2/M phase cells. However, DNA-PKcs specific inhibitor NU7026 could markedly raised G0/G1 phase cells proportion and inhibited S + G2/M phase cells proportion. Representative histograms of cell cycle alteration are shown in Fig. 3D. Meanwhile, we detected the effect of DNA-PKcs on apoptosis using TUNEL assay (Fig. 3E). Although NU7026 raised slightly TUNEL positive cells in hypoxic cells, it did not reach statistical significance.

### DNA-PKcs promoted PASMCs proliferation via interaction with NOR1

**Fig. 4:**
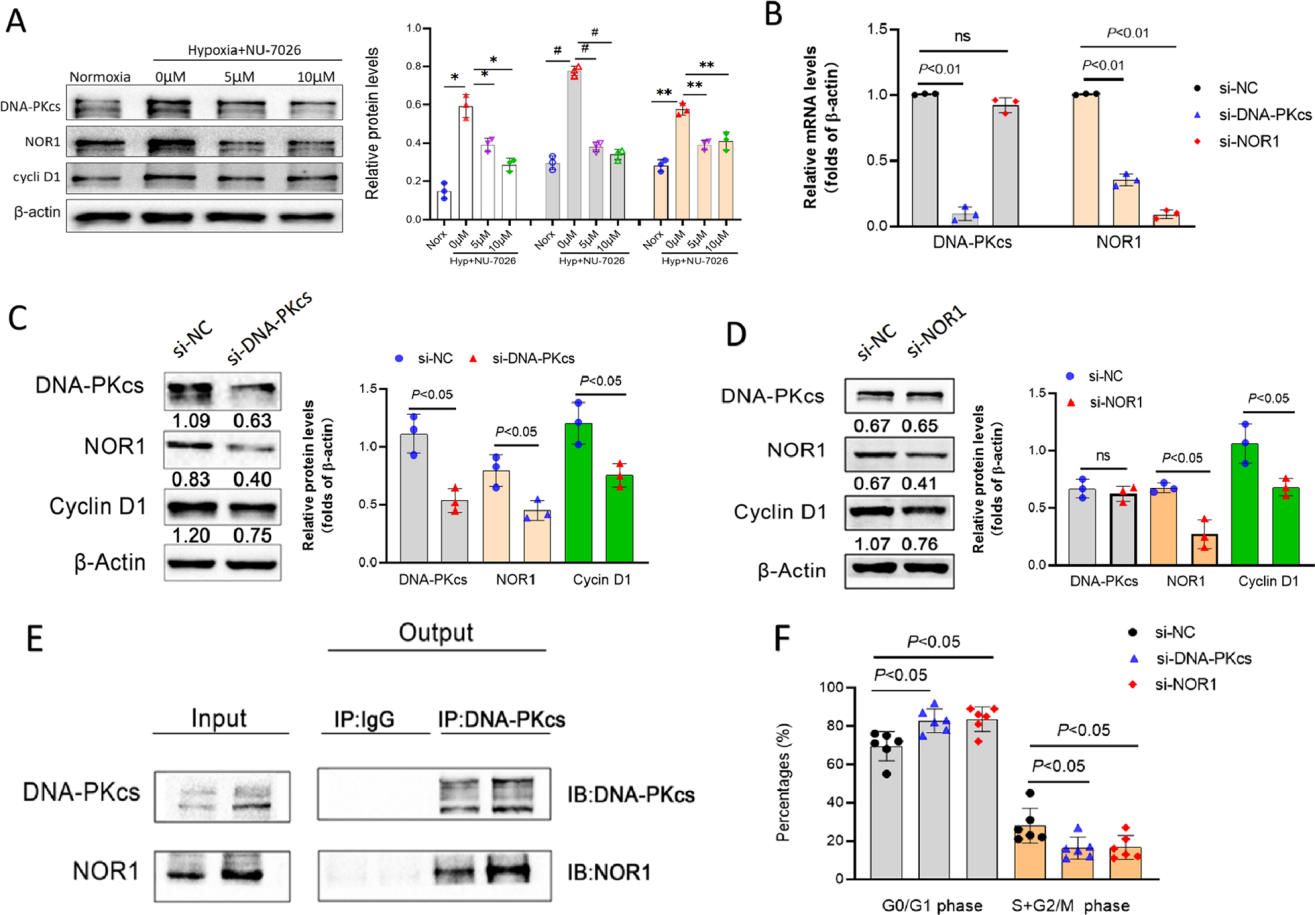
DNA-PKcs interacted with NOR1. **A** DNA-PKcs specific inhibitor (NU7026) suppressed down-stream targets in hypoxic PASMCs. **B** Affect of specific siRNA on mRNA expression of DNA-PKcs and NOR1 in hypoxic PASMCs. **C** DNA-PKcs specific siRNA inhibited down-stream targets in hypoxic PASMCs. **D** NOR1 specific siRNA inhibited down-stream targets but not affected DNA-PKcs expression in hypoxic PASMCs. **E** Co-IP showed interaction of DNA-PKcs with NOR1. **F** Specific siRNA arrested cell cycles in hypoxic PASMCs

As shown before, DNA-PKcs inhibitors markedly attenuated hypoxia-induced the decreased proportion of cells in G1 and the increased proportion of cells in S phase (Fig. 3D). And cyclin D1 is an important regulator of G1 to S-phase transition. Therefore, we speculated that DNA-PKcs affected cell cycle proteins by activating NOR1, which in turn has an effect on hypoxia-induced pulmonary vascular remodeling. We next attempted to understand the relationship among DNA-PKcs, NOR1, and cyclinD1 in hypoxia-induced pulmonary vascular remodeling. We firstly examined the protein expression of the three molecules by using western-blot after hypoxia with or without NU7026. As expected, the expression of DNA-PKcs, NOR1 and cyclin D1 were all highly upregulated by hypoxia. Meanwhile, NU7026 significantly suppressed not only DNA-PKcs levels but also the protein levels of NOR1 and cyclin D1 in hypoxia, at a dose-dependent manner (Fig. 4A).

To understand the regulation relationship between DNA-PKcs and NOR1, we utilized small interfering RNA (siRNA) to knock down the expression level of DNA-PKcs or NOR1. The interference efficiency of siRNAs was validated at both mRNA and protein levels. Although DNA-PKcs specific siRNA inhibited mRNA levels of DNA-PKcs and NOR1, NOR1 specific siRNA had no effect on DNA-PKcs mRNA levels (Fig. 4B). Additionally, we found that knockdown of DNA-PKcs prevented the increased expression of NOR1 and cyclin D1 which were induced by hypoxia (Fig. 4C). Cells transfected with siRNA against NOR1 significantly reduced protein expression of NOR1 and cyclin D1, while the DNA-PKcs protein levels have no change as compared with negative control siRNA (Fig. 4D). The results revealed that DNA-PKcs can regulate the expression of NOR1, which in turn can regulate the expression level of cyclin D1.

Then, we investigated the ability of DNA-PKcs to interact with NOR1 in human PASMCs under normoxia and hypoxia. Strikingly, coimmunoprecipitation of NOR1with DNA-PKcs was severely increased in hypoxia (Fig. 4E). Meanwhile, hypoxia promoted G1/S phase, whereas the down-regulation of DNA-PKcs and NOR1 attenuated the effects of hypoxia (Fig. 4F). Based on these results, we conclude that DNA-PKcs affects HPASMCs proliferation by regulating NOR1 protein synthesis followed by the expression of cyclin D1.

### Knockdown of DNA-PKcs prevented pulmonary hypertension in hypoxic rats

**Fig. 5:**
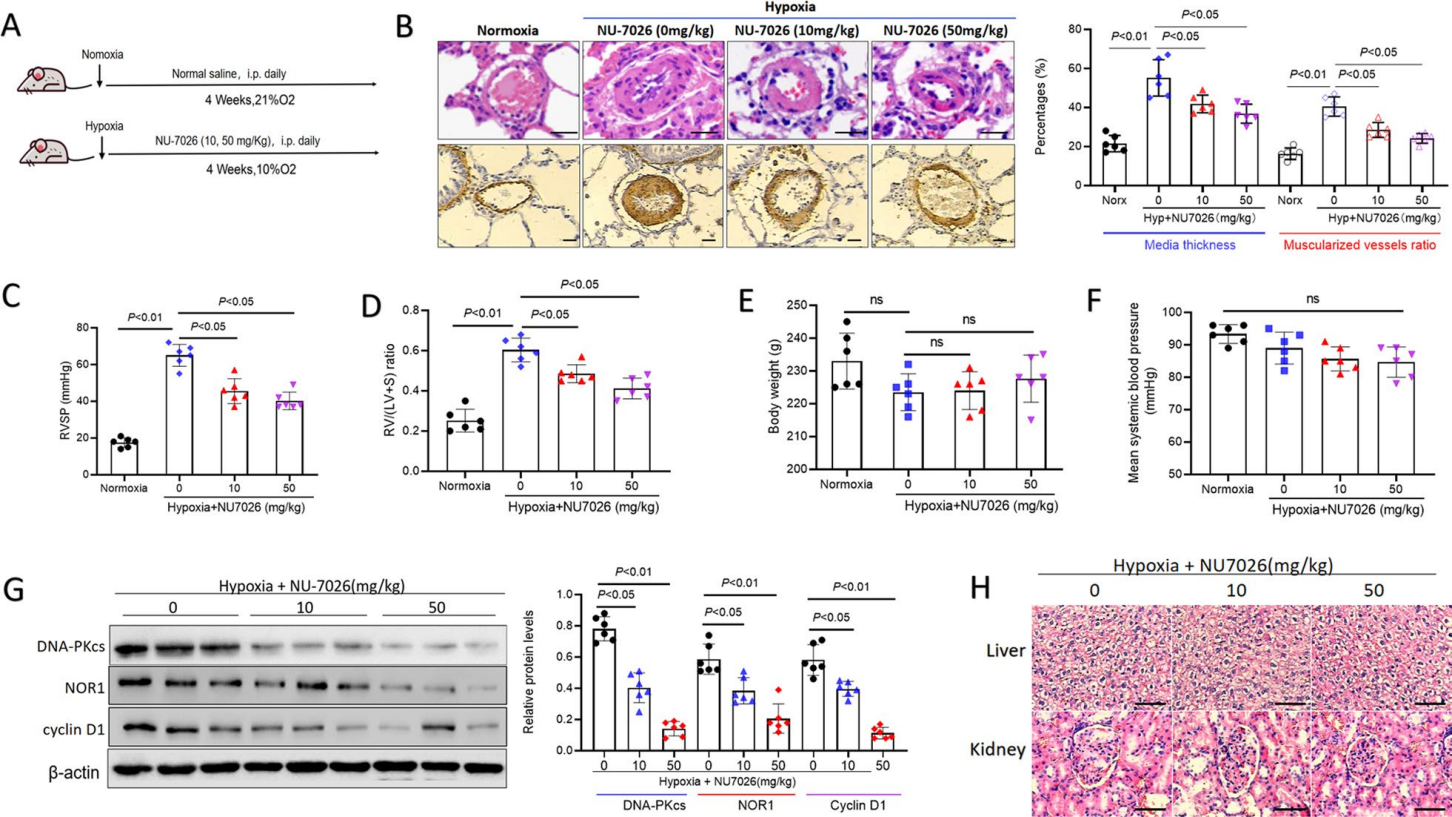
DNA-PKcs specific inhibitor (NU7026) prevented pulmonary hypertension in hypoxic rats. **A** Treatment for rat models of hypoxic PH. **B** NU7026 prevented pulmonary vascular remodeling in hypoxic rats. **C**, **D** NU7026 prevented right ventricular systolic pressure (RVSP) and right ventricular hypertrophy index [RV/(LV + S)]. **E**, **F** Changes of body weight and mean systemic blood pressure. **G** NU7026 suppressed protein levels of DNA-PKcs, NOR1 and cyclin D1 in hypoxic PH. **H** No pathological change was observed in liver and kidney after NU7026 treatment. Bar 100 µm

To further verify whether DNA-PKcs is required for the progress of HPH, we inhibited DNA damage repair using NU7026 targets to DNA-PKcs in vivo. The study flow is reported in Fig. 5A. After exposed to persistent 10% O_2_ for 4 weeks, the H&E staining and immunohistochemistry results showed that hypoxia promoted pulmonary vascular media thickness enlargement and exaggerated muscularized vessels ratio as compared with nomoxia rats (Fig. 5B). However, DNA-PKcs specific inhibitor NU7026 significantly prevented the hypoxia-induced pulmonary vascular remodeling at a dose-dependent manner, including inhibition of pulmonary arterial media thickness and muscularized vessels ratio (Fig. 5B). Furthermore, DNA-PKcs specific inhibitor NU7026 markedly abated hypoxia-induced right ventricular systolic pressure (RVSP) and right ventricular hypertrophy index [RV/(LV + S)] (Fig. 5C, D). There were no alterations in body weight or systemic hemodynamics between hypoxic and normoxic groups, regardless of treatment (Fig. 5E, F). Simultaneously, NU7026 administration not only prevented DNA-PKcs levels but also suppressed NOR1 and cyclin D1 protein levels in hypoxia exposed rats (Fig. 5G). We observed no advert effect or pathological change in liver and kidney (Fig. 5H).

### Inhibition of DNA-PKcs reversed pulmonary vascular remodeling but not pulmonary hypertension after HPH models

**Fig. 6:**
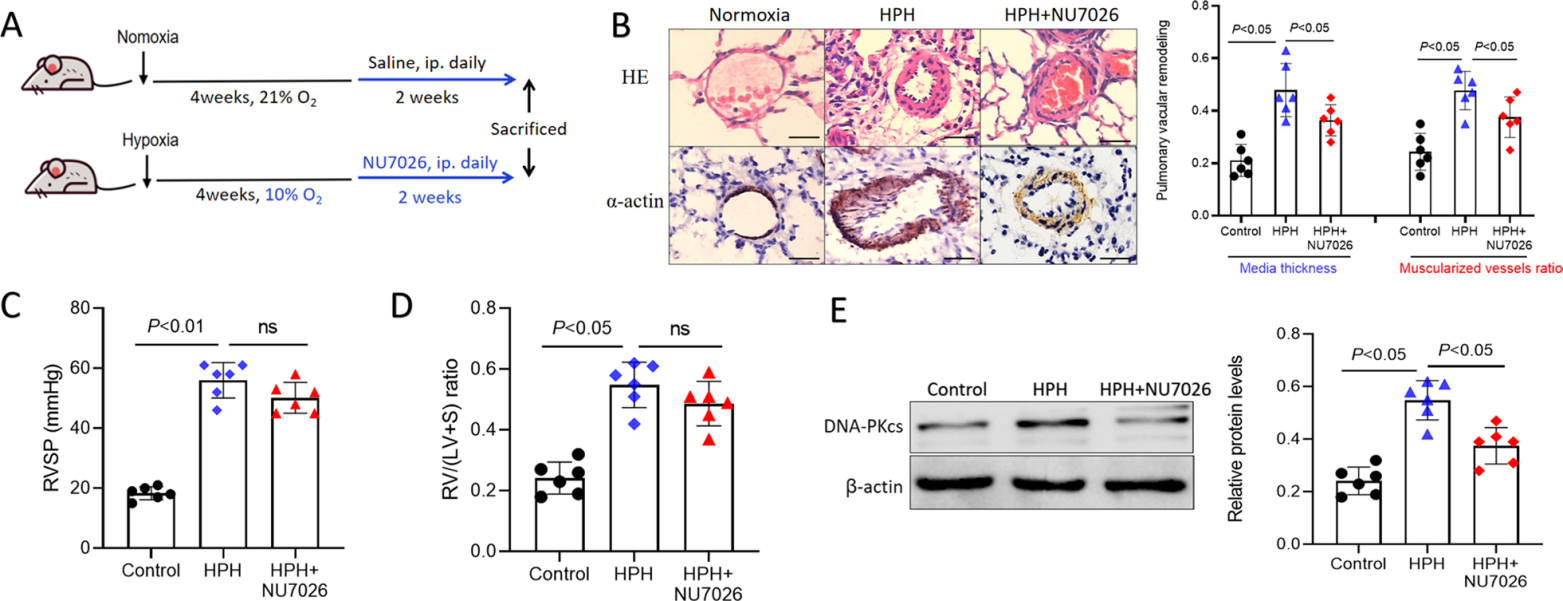
Treatment with DNA-PKcs specific inhibitor (NU7026) reversed pulmonary vascular remodeling but not pulmonary hypertension after HPH models established. **A** Establishment and treatment for rat HPH models. **B** NU7026 reversed pulmonary vascular remodeling after rat HPH models establishment. **C**, **D** NU7026 have no change on right ventricular systolic pressure (RVSP) and right ventricular hypertrophy index [RV/(LV + S)]. **E** NU7026 suppressed DNA-PKcs protein levels in rat HPH models. Bar 100 µm

To identify the value of DNA-PKcs as target of HPH treatment, HPH rats were administrated using DNA-PKcs inhibitor NU7026 after HPH models were established. The study flow is reported in Fig. 6A. Treatment of NU7026 significantly reversed the hypoxic pulmonary vascular remodeling (shown by suppression of media thickness enlargement and muscularized vessels ratio in Fig. 6B). However, NU7026 did not reverse the pulmonary hypertension and right ventricular hypertrophy [shown by RVSP and RV/(LV + S) in Fig. 6C and D]. Although NU7026 suppressed DNA-PKcs levels markedly (Fig. 6E), it only reversed pulmonary vascular remodeling, without changes of pulmonary hypertension and right ventricular hypertrophy.

## Discussion

In this study, we firstly identified the role of DNA-PKcs, a DNA repair molecule, in hypoxia PH. DNA-PKcs promoted human PASMCs proliferation via interacting with NOR1 and enhanced down-stream target cyclin D1. Inhibition of DNA-PKcs not only suppressed hypoxia-induced cells proliferation in vitro, but also reserved pulmonary vascular remodeling in HPH rat models in vivo. However, DNA-PKcs inhibition did not reversed pulmonary hypertension and right ventricular hypertrophy after HPH establishment. These results proved the role of DNA-PKcs in HPH development. But its value in HPH treatment remain need further exploration.

Because of the lack of effective curative options in HPH, there is a pressing need to explore novel therapeutic targets [1–4]. DNA damage in PAH patients has recently drawn increasing attention. To this date, there are few studies about the role of DNA repair on hypoxic pulmonary hypertension [16, 22–24]. Previous reports have identified the elevation of DNA damage markers and the role of DNA repair molecules in pulmonary hypertension, such as poly (ADP-ribose) polymerase-1 (PARP-1) and X-box-binding protein 1 [13–22]. In this research, we first explored the function of another DNA repair molecules, DNA-PKcs, in hypoxia induced pulmonary vascular remodeling. Our results confirmed the crucial role of DNA damage and repair response in pulmonary vascular remodeling, which might be a potential approach for treatment of pulmonary hypertension.

As a principal cellular internal mechanism, DNA repair plays an indispensable role in decreasing the risk of cancer and some other critical human diseases [11–19]. Being central part of the DNA repair machinery, DNA-dependent protein kinase (DNA-PK) seems to be involved in other signalling processes [20, 21]. DNA-PKcs as a part of DNA-PK is a molecular sensor for DNA damage that enhances DSB repair [20, 21]. Currently, several studies have profiled that DNA repair can affect the development of many human diseases. Recently reports indicated that DNA-PKcs acted role in multiple cancers and cardiovascular diseases, including hematological and solid tumors, cardiac ischemia reperfusion [36, 37]. DNA-PKcs expression and activity are frequently deregulated in multiple hematological and solid tumors and have been tightly linked to poor outcome [36]. DNA-PKcs serves as a novel causative factor for mitochondrial damage via suppression of Bax inhibitor-1, en route to the onset and development of cardiac ischemia reperfusion injury [37]. DNA-PKcs were observed promoting proliferation of human aortic vascular smooth muscle cells (VSMC), leading to neointimal formation [35]. However, we still have no direct evidence to prove that DNA-PKcs has an association with hypoxia PH at present. In our study, we firstly demonstrated that the expression of DNA-PKcs is upregulated under hypoxia and DNA-PKcs functions to promote proliferation of human PASMC in vitro and in vivo. Furthermore, we confirmed that DNA-PKcs acted role via interacting with NOR1, an immediate-early responsive gene which had been proved participating in pulmonary vascular remodeling in our previous reports [31–33]. Hence, these results might enrich the underlying mechanism of DNA damage repair in hypoxic PH and provide a potential therapeutic target for PH.

NOR1 is an immediate-early responsive gene that responds to various extracellular stimuli including hypoxia [25, 26]. Several studies have proved that NOR1 is significant to the proliferation of VSMC [28–30]. Our previous and recent studies also revealed that NOR1/cyclin D1 pathway promoted PASMCs proliferation in the process of pulmonary vascular remodeling and PH [31–33]. In this study, the results demonstrated again that NOR1 could stimulate PASMCs proliferation and pulmonary vascular remodeling through the regulation of cyclin D1 protein. Moreover, we illustrated the mechanistic interaction between DNA-PKcs and NOR1 in hypoxic PH by using co-immunoprecipitation assays. As expected, we found that the ability of DNA-PKcs to interact with NOR1 in human PASMCs was grossly increased in hypoxia. Additionally, we verified the effect of specific blockage of the DNA-PKcs by using the small molecular inhibitor NU7026. Our results indicated that NU7026 alleviated hypoxia- and monocrotaline- induced pulmonary vascular remodeling in vivo.

However, we should note that our findings confirmed only that pulmonary vascular remodeling requires DNA-PKcs. The exact mechanism underlying the DNA repair occurring in HPH remains to be ascertained. Cells have multiple pathways to repair particular types of DNA damage. We can not exclude other DNA repair pathways are involved. We also cannot rule out the potential involvement of other DNA repair factors. We look forward to further studies that may confirm the main associated DNA repair factors.

In conclusion, this is the first time to reveal that DNA-PKcs pathway plays a critical role in hypoxic pulmonary vascular remodeling and HPH. Meanwhile, our study showed the potential mechanism of DNA-PKcs and NOR1 in the development of HPH. Therefore, this report might enrich the underlying mechanism of DNA damage repair in HPH and provide a potential therapeutic target for PH.

## Data Availability

The datasets used and/or analyzed during the current study are available from the corresponding author on reasonable request.

## Abbreviations

HPH: Hypoxic pulmonary hypertension
PASMC: Pulmonary artery smooth muscle cells
Co-IP: Co-immunoprecipitation
CCK-8: Cell counting kit-8 assay
DDR: DNA damage response
DSB: DNA double-strand breaks
NHEJ: Non-homologous end joining
FBS: Fetal bovine serum
PI: Propidium iodide
PBS: Phosphate-buffered saline
FEV1/FVC: Forced expiratory volume in 1 s/fixed vital capacity ratio
